# Evaluating a Standardized Transition of Care Process for Pediatric Inflammatory Bowel Disease Patients

**DOI:** 10.3390/children7120271

**Published:** 2020-12-04

**Authors:** Jennifer Lynne Shearer, Sharon Perry, Nicole Lidyard, Carolyn Apperson-Hensen, Sarah DeLozier, Kimberly Burkhart, Jeffry Katz, Jonathan Moses

**Affiliations:** 1 School of Medicine, Case Western Reserve University, Cleveland, OH 44106, USA; jls403@case.edu (J.L.S.); cva9@case.edu (C.A.-H.); 2 UH/Rainbow Babies Children’s Pediatric Gastroenterology, University Hospitals Cleveland, Cleveland, OH 44106, USA; Sharon.perry@uhhospitals.org (S.P.); Nicole.lidyard@uhhospitals.org (N.L.); 3 Center for Clinical Research, University Hospitals Cleveland, Cleveland, OH 44106, USA; Sarah.delozier@uhhospitals.org; 4 UH/Rainbow Babies and Children’s Pediatric Psychology, University Hospitals Cleveland, Cleveland, OH 44106, USA; Kimberly.Burkhart@uhhospitals.org; 5 Division of Gastroenterology, University Hospitals Cleveland Medical Center, Cleveland, OH 44106, USA; jeffry.katz@uhhospitals.org

**Keywords:** inflammatory bowel disease, pediatrics, young adults, transition, multidisciplinary

## Abstract

To reduce lapses in care for pediatric inflammatory bowel disease (IBD) patients approaching adulthood, a health maintenance transition visit (HMV) was developed to supplement standard medical care (SMV). Our aim was to assess the effect of the HMV on transition readiness. A retrospective chart review was conducted at a single center with demographics and clinical data from HMV and SMV visits. Effectiveness of the HMV was assessed by the patient health questionanaire-9 (PHQ-9) and transition readiness assessment questionnaire (TRAQ) scores. A total of 140 patients, 80% Caucasian and 59% male completed an HMV. The mean age was 18 ± 2 years old, and 93% of patients reported inactive or mild disease. Patients who completed at least 1 prior HMV scored significantly higher on the TRAQ when transferring to adult care compared to patients transferred at their first HMV visit (92 vs. 83, *p* < 0.05). Of patients with no prior depression diagnosis, 36% had a positive screen for depression. A significant relationship was identified between disease status and PHQ-9 (*p* < 0.05). This study demonstrated a structured HMV increased transition readiness and quantified the significant under-diagnosis of depression in this population, emphasizing the importance of screening. These results indicate depression may affect patients’ transition preparedness.

## 1. Introduction

The incidence of pediatric-onset inflammatory bowel disease (IBD), including Crohn’s disease and ulcerative colitis (UC), is rising worldwide [1,2]. Estimates suggest 25% of patients with IBD presented before 20 years of age [3]. Pediatric-onset IBD tends to demonstrate both extensive disease and a rapid progression such that patients require early immunomodulating therapy [3]. However, the impact of IBD on an adolescent patients’ quality of life extends beyond their standard medical care. Adolescent patients with IBD are at an increased risk of depression and poor adherence to treatment plans [4,5,6]. This poor adherence exacerbates disease activity, results in lapses in care, and predisposes patients to preventable complications [7].

Transition is “a purposeful planned movement of adolescents and young adults with chronic conditions from child-centered to adult-oriented health care systems” [8]. However, this transition is two-fold, including both the transition from pediatric to adult providers and the gradual transition of responsibilities from parent to patient [9,10]. A recent survey suggests 80% of adult gastroenterologists found their young adult patients were inadequately prepared for transition to adult care [11]. One study found only 5.6% of IBD patients on the verge of their transitions to adult care met the institutional benchmark for transition readiness [12].

Several transition models have been proposed to integrate efforts by the provider, the patient, and the family towards a timely and appropriate transition, which include a transition coordinator, a transition program integrated into standard care visits, and independent transition clinics [9]. Despite the conceptual development of these interventions, no clear data has supported one transition model as more effective than other models of care. In 2014, the University Hospitals Rainbow Babies Pediatric Gastroenterology Department initiated a multi-disciplinary Pediatric IBD Health Maintenance Clinic. This clinic offered a health maintenance visit (HMV) to supplement the standard of care medical visits (SMV) and to prepare patients for the transition process. The aim of this study is to evaluate the efficacy of this structured HMV and its utilization of several screens to assess patient readiness for transition into adult care.

## 2. Materials and Methods

### 2.1. Clinic Development and Description

**Figure 1:**
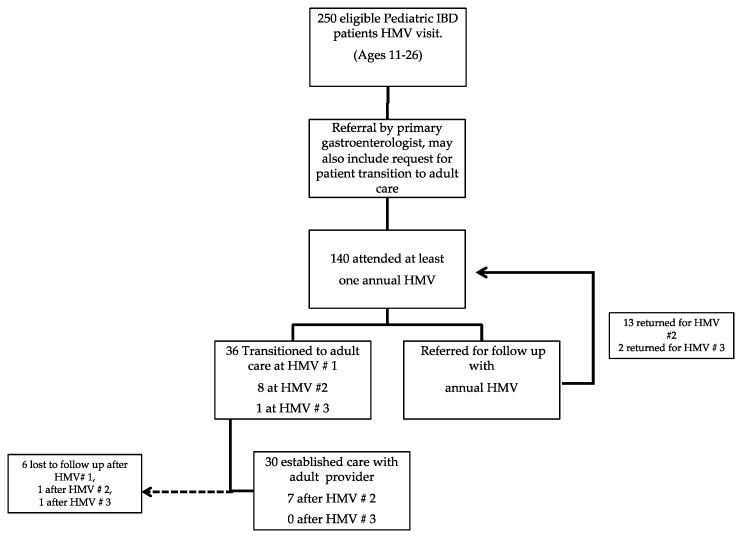
Patient Flow through Health Maintenance visits (HMV) and Transition of Care visits. IBD: inflammatory bowel disease. Number of HMV visits completed indicated by # followed by numeral.

This study is a retrospective chart review of patients seen at the IBD Health Maintenance Clinic at a large pediatric academic medical center in the United States. Since 2014, 140 patients were seen at the clinic. Participation in the clinic included an annual health maintenance visit (HMV) to supplement standard pediatric gastroenterology visits (Figure 1). Patients, aged 11–26 years, were referred for an HMV by each patient’s primary gastroenterologist.

Additionally, patients deemed ready to transition by each patient’s primary gastroenterologist would be transferred to adult gastroenterology at a transition specific HMV. Patients completing a transition-HMV were expected to be seen by their new adult gastroenterologist within 6–8 weeks of this transfer. If the patient was not deemed appropriate for transition by the primary gastroenterologist, they were scheduled for another HMV within 1–2 years. This was an iterative process until the primary gastroenterologist deemed them ready for transition to adult gastroenterology.

Visits included a comprehensive review of patients’ medical history, targeted transition goals, disease-specific health maintenance expectations (i.e., vaccinations, colonoscopies, infection screening, and bone density scans) by the nurse educator and specialized IBD nurse practitioner or physician [13]. Finally, patients also completed two validated health screenings: the transition readiness questionnaire (TRAQ) and patient health questionnaire 9 (PHQ-9). The TRAQ questionnaire was reviewed and discussed with patient and parents by IBD specialized provider and nurse educator to develop targeted, patient specific transition goals. These goals centered around four common themes including IBD education, adherence, transition of responsibility, and the logistics of transferring care, along with assessment of disease impact on mental health and behavioral health treatment recommendations. Targeted transition goals were then reviewed at subsequent HMV follow up visits.

As part of the HMV, patients met with a multi-disciplinary team including a nurse educator, a specialized IBD nurse practitioner or physician, dietitian, social worker and psychologist. Patients were seen independently by each provider at the start of the visit which entailed review of detailed medical summary with targeted education based on the patient’s clinical history. The IBD nurse educator spent time reviewing age appropriate self-management skills and discussion of acquisition of new skills prior to the next HMV. The social worker reviewed the patient’s school accommodations and insurance coverage to help address gaps in resources. Psychological support included review of PHQ-9 and appropriate referrals with follow up as needed. The dietician performed a formal review of growth parameters, discussed patients’ dietary needs and nutritional balance, vitamin supplementation, and weight management.

### 2.2. Transition Readiness Assessment

The TRAQ is a patient-completed 20-item questionnaire, which assesses the patient’s disease self-management and health care utilization skills (DOI: 10.1016/j.acap.2014.03.008) [14]. The questionnaire utilizes a 5-point Likert scale. Patients rate their ability level on a variety of tasks including medication adherence, communication with medical providers, and knowledge of insurance coverage. In addition to scoring individual questions, a summative score, 0–100 possible, and a count of mastered skills, those which received score of 5. A higher summation score indicated greater readiness. Since 2014, all patients greater than 14 years of age were asked to complete a TRAQ annually at his/her HMV. Parental presence and/or assistance was noted on each questionnaire was assessed by the provider after completion and recorded.

### 2.3. Depression Screen

The PHQ-9 is a self-administered depression screener [15]. Each of the 9 Diagnostic and Statistical Manual of Mental Disorder (DSM-IV) criteria is scored as “0” (not at all) to “3” (nearly every day). The total score is then interpreted as depression severity from minimal to severe [15].

### 2.4. Disease Activity

Disease activity was assessed using the pediatric ulcerative colitis activity index (PUCAI) and the short pediatric Crohn’s disease activity index (PCDAI), which were calculated from data collected from the patient’s last standard care visit [16,17,18]. This standard of care visit was required to be within 3 months of the patient’s HMV. The physician global assessment (PGA) disease status was also recorded as determined by the patient’s primary gastroenterology provider based on the patient’s recent symptoms (abdominal pain, number of stools, weight), physical exam findings, laboratory studies, and general well-being [16].

### 2.5. Data Source

Data was sourced from the clinic’s electronic health record. Data was stored in Research Electronic Data Capture (REDCap) [19,20]. The database included 140 patients. Data reflects patient presentation at HMV visits and pre/post standard of care visits (labs and visit note). Patient demographic information (age, ethnicity), past medical history, disease status, and medications were sourced from visit notes. All patients seen for an HMV were included in the study except for patients with diagnosis of Down syndrome or another development delay.

### 2.6. Statistical Analysis

Statistical analysis was performed via SAS Version 9.4 (SAS Institute, Cary, NC, USA) and SPSS Version 25.0 (Armonk, NY, USA: IBM Corp.). All statistical tests with a *p*-value < 0.05 were considered significant. Mean ± SD were reported for all continuous variables that followed a Gaussian distribution such as age and TRAQ. A *t*-test was performed to compare differences of continuous variables. Categorical variables were reported as count (percentage), including primary diagnosis and sex. Outcomes were compared with chi-square tests and Spearman rank-order correlations.

## 3. Results

### 3.1. Patient Characteristics

A total of 140 patients who completed at least 1 HMV since the transition clinic began in 2014 were included in this study. Our cohort was 59% male and 80% white (Table 1). At their first HMV, patients’ ages ranged from 11 to 26 years old but the average age was 18 ± 2 years. On average, patients were diagnosed with IBD at the age of 13 ± 5 years. The majority of patients (75%) had a primary diagnosis of Crohn’s disease. Additionally, 40% of patients had a secondary diagnosis, including anxiety, depression, or gastroesophageal reflux disorder (Table 1). Based on each patient’s PUCAI or PCDAI at the most recent standard care visit, 93% of patients had inactive or mild disease at the time of their HMV.

(Table 1). Only one patient returned to pediatric gastroenterology after this initial transfer due to insurance limitations. The patients who were not transferred to adult care (*n* = 104) were recommended to continue with their pediatric gastroenterologist and return for a follow up HMV in one year.

### 3.2. PHQ-9 Depression Screen

**Figure 2:**
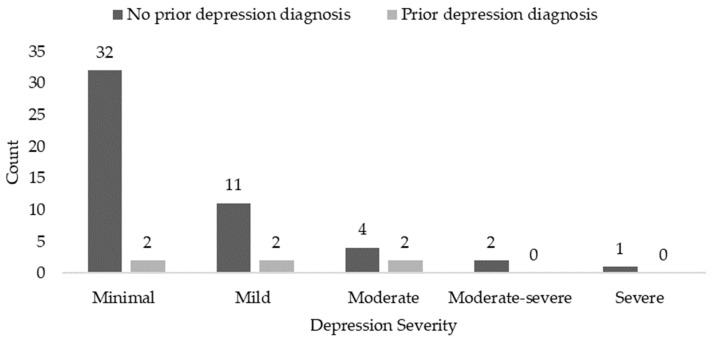
Patient health questionnaire 9 (PHQ-9) depression screen by prior depression diagnosis.

The PHQ-9 screen was administered to 56 patients at their HMV. Only 6 of these patients had a prior diagnosis of depression. Of the patients with no prior depression diagnosis (*n* = 50), 36% of patients had a positive depression screen (Figure 2), ranging from mild to severe depression.

Questionnaires were then reviewed with a social worker or/and psychologist with referrals to a psychosocial health profession, as appropriate, and an overview of evidence-based treatment was provided.

No significant correlations were found between the PHQ-9 and the PUCAI score (*p* = 0.154), but a significant correlation was found between the PHQ-9 and the short PCDAI, such that higher scores on the PHQ-9 were significantly associated with higher scores on the short PCDAI (*p* = 0.011). A significant relationship was identified between PGA and PHQ-9 scores such that patients with inactive disease (*n* = 10) were more likely to report only minimal depression (1–4 points) than patients with mild disease (*n* = 3), (*p* < 0.05), and patients with mild disease were more likely to report mild depression (5–9 points) than patients with inactive disease (*p* < 0.05). PHQ-9 scores were also negatively correlated with final TRAQ scores, such that higher levels of depression were related to lower TRAQ scores (*p* < 0.05). PHQ-9 scores were not associated with clinical determination by providers to transition to adult care.

### 3.3. Transition Readiness Assessment

The TRAQ was administered to all patients 14 years and older. In total, 111 questionnaires were completed at the first HMV visit. Patients were instructed to complete the questionnaire on their own. However, it was noted if parent was present and/or aided in the completion of the questionnaire. Across all patients the average TRAQ score was 66 ± 17. Scores on the TRAQ score were negatively correlated with scores on the PHQ-9, such that lower TRAQ scores were associated with higher PHQ-9 scores (*p* = 0.021). TRAQ scores were also positively correlated with age at transition visit, such that TRAQ scores were higher for older children (*p* < 0.001). TRAQ scores were also higher for children whose parents were not present, *p* < 0.001. No significant relationships were found between TRAQ scores and ethnicity (*p* = 0.120), PUCAI scores (*p* = 0.911), short PCDAI scores (*p* = 0.193), or sex (*p* = 0.186).

Of the patients transferred to adult care at their first HMV, the average TRAQ score was 83 ± 12 compared to 64 ± 15 for those who were continuing with pediatric care (*p* < 0.01). In addition to the difference between the summed TRAQ scores, trends and differences between these two follow up groups were also noted by TRAQ specific questions. In regards to the question “do you answer questions that are asked by the doctor, nurse, and clinic staff?” both patients continuing in with pediatric care and those transitioned to adult care predominantly scored 4s and 5s. There was no significant difference between these groups. However, other questions such as “do you call the doctor about usual changes in your health?” revealed significant differences (*p* < 0.01) between the two follow up groups scoring trends. While the patients continuing in pediatrics trended towards scoring 1s and 2s (towards the left), the patients transitioning to adult care were skewed towards 4s and 5s (towards the right).

**Figure 3:**
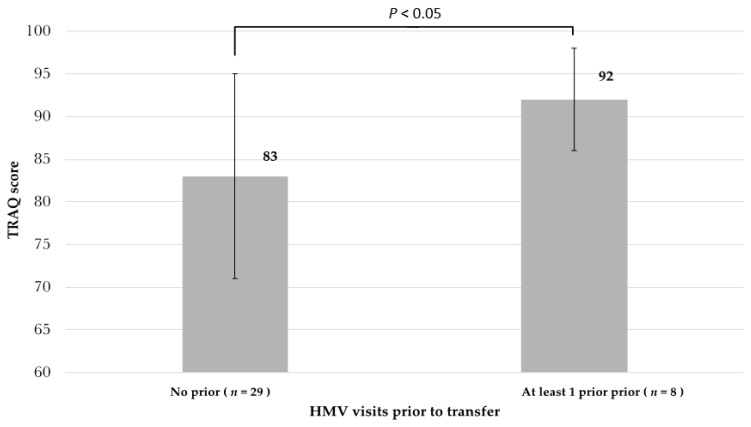
Average transition readiness assessment questionnaire (TRAQ) at time of transfer based on number of HMV attended.

Of the 13 patients who returned for a follow up HMV, 9 patients were then transferred to adult care, i.e., at second or third HMV. TRAQs were administered at all follow up HMVs. Despite no difference in disease severity nor age, patients who completed at least one HMV prior to the HMV-transition visit (i.e., completed a total of 2 or more visits at the time of transfer) had a statistically high TRAQ score at the time of transfer (92 vs. 83, *p* < 0.05, Figure 3).

## 4. Discussion

This study evaluated the clinical application of a specialized multi-disciplinary health maintenance and transition readiness clinic for pediatric patients with IBD. Patients’ transition readiness, assessed via the TRAQ questionnaire, displayed age-associated increases in health-related skill acquisition during the transition period. Our results also suggest patients who completed at least one prior HMV before their HMV-transition visit scored significantly higher on the transition readiness assessment. Finally, utilization of the PHQ-9 depression screen revealed a substantial under-diagnosis of depression in this vulnerable young adult population. The identification and treatment of depression may in turn improve disease management [21].

Since gaining awareness across medical specialties, several transition models have been proposed to support pediatric patients through the transition from pediatric to adult providers. These models include transition coordinators, programs integrated into standard care visits, and, as evaluated in this study, a specialized transition clinic. However, the clinical application of these models is still limited. This study of 140 patients seen in a specialized transition clinic is the largest study of adolescent IBD patients in the transitional period to date and includes patients at different stages along the spectrum of transitional preparations.

Across transition of care models, the validated TRAQ has been utilized to assess patients’ skill acquisition and preparedness. Still, there is a noticeable gap between department benchmarks for transition and the reality of patient preparedness [22]. Due to the longitudinal nature of the HMV visit, beginning as young as 11 years of age, this study also provides insight into the progression of the skill acquisition, estimated by the TRAQ, over time. While age was the only variable correlated with the summative TRAQ score, more detailed analysis revealed specific similarities and, more importantly, differences between those patients deemed prepared to transition to adult care and those continuing with pediatric providers. For example, both patient populations reported similar scores when asked if they respond to questions from their providers—a prompted participation. However, patients transitioning to adult providers were significantly more likely to call their doctor about changes in their health—an unprompted participation. These results suggest specific actions or types of “unprompted” engagement in the healthcare systems make serve as an important marker or goal when preparing to enter adult care. As patients that completed at least one addition HMV prior to their transitioning-HMV visit had a significantly higher TRAQ score, it appears participation in a transitional care program, may improve this skill acquisition and improve patient’s readiness for adult care.

Similar to the growing awareness of transition in general, the increasing prevalence of depression in the young adult IBD population is also gaining recognition [23,24]. Few studies have assessed if this recognition has affected the screening, diagnosis, and intervention in the clinical setting. The administration of the PHQ-9 depression screen at the HMV visit not only revealed an overall under-diagnosis of depression but also suggests that those “missed” in standard care depression evaluations crossed the spectrum of depression severity from mild to severe. The HMV setting also offered patients a unique opportunity for both screening and intervention during a single visit. While the standard care visit is limited due to time constraints and diverting or distracting acute disease processes, the inclusion of the depression screen in a separate transition-oriented HMV allowed the time to assess the patient’s longitudinal physiologic and psychologic well-being and allowed for immediate intervention with the multidisciplinary social work and psychology teams without a delay or need for referral. These results emphasize the importance of screening young adult patients with IBD to assess transition readiness and psychological status prior to transition.

There were several limitations to this study due to its inherent retrospective nature. Additionally, as there is no objective, validated tool to determine when to transition a patient to an adult provider, this determination was made subjectively by each patient’s primary gastroenterologist. The screening tools administered were also based on self-reporting. Patients were instructed to complete each questionnaire independently; however, parent’s presence may have influenced patient reporting. To minimize this effect, parent presence and involvement was noted on each questionnaire and analysis revealed there was no statistically significant correlation between parental presence and TRAQ scores. Although well validated in adolescents and young adults with chronic illnesses requiring transition of care to adult providers, the TRAQ was not specifically validated in pediatric IBD, although there are previously published reports utilizing this screening tool [9,22,25,26]. Controlling for all factors that could have influenced the TRAQ scores and our results were an additional limitation and likely due to small patient sample size. Finally, due to the recent development of the clinic, there was limited follow up and longitudinal assessment of patient functioning in the adult clinical setting.

In conclusion, this study emphasizes the importance of screening pediatric patients to assess both transition readiness and psychological status prior to transition to adult care. Our results also suggest a structured multi-disciplinary approach to IBD transition may improve patients’ readiness. Future studies will need to evaluate patient and parent perspectives and satisfaction of this clinical experience and the utility of the TRAQ to determine the appropriate timing of transition.

## Figures and Tables

**Table 1:** Clinical Characteristics of Patients at First HMV.

Demographics	*n* = 140
Age (years) at Initial HMV	18 ± 2
Age (years) at Diagnosis	13 ± 2
White [*n* (%)]	112 (80)
Male [*n* (%)]	83 (59)
Primary Diagnosis	
Crohn’s Disease	103 (75)
Ulcerative Colitis	29 (21)
Unclassified	6 (4.3)
Secondary Diagnosis	
At Least One Secondary Diagnosis *	56 (40)
Anxiety/Depression	30 (21)
GERD	15 (11)
Other	44 (31)
None	83 (60)
Recommended Follow Up Course	
Transitioned to Adult	36 (26)
Return annual HMV	114 (74)
both diagnoses)	
HMV = Health Maintenance Visit	
GERD = gastroenterology reflux disease	
Data are number (percent) of patients or mean ± SD	

Over a quarter of patients (*n* = 36) were transferred to adult gastroenterology at their first HMV; *: Patients with multiple secondary diagnoses (ex: depression and GERD were counted under both diagnoses).
